# Budget Impact Analysis of Pharmacist-Led Medication Management in Cardiovascular and Type 2 Diabetic Patients

**DOI:** 10.3390/healthcare10040722

**Published:** 2022-04-13

**Authors:** Iva Mucalo, Andrea Brajković, Marija Strgačić, Djenane Ramalho-de-Oliveira, Elizabeta Ribarić, Ana Bobinac

**Affiliations:** 1 Centre for Applied Pharmacy, Faculty of Pharmacy and Biochemistry, University of Zagreb, 10 000 Zagreb, Croatia; abrajkovic@pharma.hr; 2 Faculty of Pharmacy and Biochemistry, University of Zagreb, 10 000 Zagreb, Croatia; mstrgacic@student.pharma.hr; 3 College of Pharmacy, Centre for Pharmaceutical Care Studies, Federal University of Minas Gerais, Belo Horizonte 31270-901, Brazil; djenane.oliveira@gmail.com; 4 Center for Health Economics and Pharmacoeconomics (CHEP), Faculty of Economics and Business, University of Rijeka, 51 000 Rijeka, Croatia; ana.bobinac@efri.hr (E.R.); elizabeta.ribaric@efri.hr (A.B.)

**Keywords:** budget impact analysis, comprehensive medication management services, pharmacists’ services, polypharmacy, medication therapy management, cardiovascular diseases, type 2 diabetes mellitus

## Abstract

The paper aims to identify and measure the costs and savings associated with the delivery of Comprehensive Medication Management (CMM) services in Croatia in patients diagnosed with hypertension accompanied by at least one additional established cardiovascular disease (CVD) and/or type 2 diabetes mellitus (DMT2) who use five or more medicines daily. The budget impact analysis (BIA) employed in this study compares the total costs of CMM to the cost reductions expected from CMM. The cost reductions (or savings) are based on the reduced incidence of unwanted clinical events and healthcare service utilisation rates due to CMM. The BIA model is populated by data on medication therapy costs, labour, and training from the pilot CMM intervention introduced in Zagreb’s main Health Centre, while relevant international published sources were used to estimate the utilisation, incidence, and unwanted clinical events rates. Total direct costs, including pharmacists’ labour and training (EUR 2,667,098) and the increase in the cost of prescribed medication (EUR 5,182,864) amounted to EUR 7,849,962 for 3 years, rendering the cost per treated patient per year EUR 57. CMM is expected to reduce the utilisation rates of healthcare services and the incidence of unwanted clinical events, leading to a total 3-year reduction in healthcare costs of EUR 7,787,765. Given the total CMM costs of EUR 7,849,962, CMM’s 3-year budget impact equals EUR 92,869, rendering per treated patient an incremental cost of CMM EUR 0.67. Hence, CMM appears to be an affordable intervention for addressing medication mismanagement and irrational drug use.

## 1. Introduction

With the increasing incidence and prevalence of chronic diseases, the demand for healthcare services is growing worldwide, exerting major funding pressures on constrained healthcare resources. Medicines are among the most common medical interventions for the treatment, prevention, and therapy of chronic diseases [1]. The demand for medicines and therefore the pharmaceutical spending is increasing worldwide, typically at rates higher than the growth rates of other health spending categories, driving the growth in total healthcare expenditure [2]. Croatia is no exception—between 2014 and 2018 the pharmaceutical spending increased on average 5% a year, while the growth of other health spending categories was slower [3]. Countries apply various pricing and reimbursement policies to curb the growth in pharmaceutical spending and contain costs (as well as increase the overall cost-effectiveness of pharmaceutical spending). However, once the reimbursement process is finished, and the medicines are listed and available to patients (with or without copayments), the procedures which monitor different aspects of postlisting follow-up of medicines, including rational prescribing and rational use monitoring, are not always in place or are underdeveloped, as is the case in South-Eastern Europe [4].

In Croatia, primary care physicians prescribe all outpatient medicines. The Croatian health insurance fund (CHIF), the main healthcare payer, strictly controls physicians’ prescribing behaviour, imposing fines if physicians do not comply with prescribing restrictions. To promote rational prescribing, CHIF’s restrictions determine which medicines can be prescribed for which diagnosis (Due to reference pricing, there is little pressure to prescribe generics). However, other aspects of rational prescribing (such as duplication of therapies, potential adverse drug events (ADEs), subtherapeutic dosage, and variations between prescribers) typically remain under the radar. Like rational prescribing, the rational use of prescribed medicines (such as monitoring polypharmacy in elderly patients and nonadherence) is also not promoted, in spite of considerable costs of inappropriate prescribing, ADEs, and nonadherence [5,6,7,8,9,10,11,12,13,14].

Comprehensive Medication Management (CMM) services provided by trained pharmacists can bridge this gap by increasing rational drug use, improving the prescribing of medicines, and reducing the unnecessary and often harmful use of medications and the resulting complications [15,16,17,18,19]. Grounded in the practice of pharmaceutical care [20,21,22] and promoted by major professional organizations [19,23,24,25], the standardised and internationally recognised CMM protocol proposed by Cipolle at all [22] is an effective approach to resolving drug therapy problems (DTPs), improving clinical outcomes [16,17,26,27,28,29,30,31,32,33], reducing costs [16,34,35], and improving patient and provider experience [16,36,37], hence increasing the value of medicines used. 

The CMM protocol of Cipolle et al. [22] targets patients with diabetes type 2 (DMT2) and/or cardiovascular diseases (CVD) because these are among the most prevalent and costly chronic diseases worldwide, with CVD being the leading cause of global mortality [38]. Croatia is no exception. Ischaemic heart disease and stroke are the two main causes of death in Croatia. The preventable mortality rates from ischaemic heart disease and stroke are twice the EU average [39]. Unlike most EU countries, the mortality rate from ischaemic heart disease decreased only slightly between 2000 and 2016, while mortality rates from diabetes have increased sharply since 2000. The rise in mortality from treatable conditions such as diabetes should be a cause for concern and an argument for introducing CMM services. The same can be said for polypharmacy, a common occurrence in the elderly and chronically ill, which increases the risk of medication errors and DTPs, namely omissions, duplicate prescriptions, and harmful interactions. In the era of aging populations, polypharmacy, multiple chronic conditions, and complex and decreasingly manageable therapy regimens, CMM programmes are especially important for chronic elderly patients taking five or more medicines, who are at an increased risk of experiencing medication errors, ADEs, duplications of therapy, and detrimental interactions and who often fail to reach therapy goals (Although other pharmacist interventions, besides CMM, also have a positive impact on patient therapy goals, various studies have demonstrated the positive impact of CMM on the management of chronic diseases, by improving individual cardiovascular risk factors such as blood pressure [30,32,33], glycosylated haemoglobin (HbA1c) [17,26,30,31,32], and LDL cholesterol [16,17,31,32]). In turn, CMMs’ data could help payers to develop increasingly detailed prescribing guidelines and update their policies to monitor and enforce rational use, which would have a potential double benefit: fewer adverse events and lower overall prescribing costs.

In January 2018, a standardised CMM service was introduced as a pilot project in the largest county health centre in Croatia—Health Centre Zagreb Centre [40], making it the first health centre in Croatia and South-Eastern Europe to offer CMM. CMM was offered to eligible patients free of charge. The CMM patient care process followed Cipolle et al. [22] methodology, as described in Table 1. The same standardised CMM service protocol was previously applied in the US [16,20,26,28] and elsewhere [27,29,30,31], in the same patient groups (i.e., patients with DMT2 and/or CVD, as explained later on). These CMM services have demonstrated their ability to improve clinical outcomes [16,17,26,27,28,29,30,31,32,33] and reduce costs [16,34,35]. However, it is unclear to what extent such standardised CMM interventions can be deemed affordable.

Budget impact analysis (BIA) assesses the affordability of interventions and helps policymakers decide whether the adoption of a new health intervention is within their means, given the resource and budget constraints of the context. So far, quantitative cost analyses and evaluations of pharmacist interventions have been in short supply [9], and the question of CMM’s affordability remains unanswered. This paper reports the results of the BIA of CMM in the Croatian context to show whether, from the payer’s perspective (Croatia operates a single healthcare payer system, the Croatian health insurance fund (CHIF), which finances and contracts all public health services), introducing a nationwide CMM is affordable. Using data from various sources, the BIA identifies and models the costs, the savings, and the nonmonetary benefits [41,42,43] associated with introducing and rolling-out a standardised nationwide CMM service [22] in Croatia over a 3-year period (2022–2024) to predict CMM’s financial impact on the CHIF’s budget. Our study contributes to the literature by being the first budget impact analysis of CMM. As such, this study adds to the small body of literature by being among the few quantitative analyses and evaluations of pharmacist interventions more generally [9]. 

## 2. Materials and Methods

### 2.1. Data Formation

The pilot CMM intervention introduced in Zagreb’s main Health Centre has provided a myriad of data [40], including the data on medication therapy costs, labour, and training. However, the data on health-related benefits of CMM from the pilot study are not mature or comprehensive enough to feed the entire BIA model. More generally, readily available data on, e.g., the rate of healthcare service utilisation or the incidence of particular clinical events does not exist in Croatia. Hence, conducting a quantitative assessment of CMM in Croatia, and other jurisdictions with similar data insufficiencies (such as South-Eastern Europe [4]), usually requires the transfer of data on incidence, health-related benefits, or outcomes from other (international) sources and studies conducted in other jurisdictions, as is also common in other economic assessments (e.g., HTA). 

A large-scale US study by Ramalho de Oliveira et al. (2010) was used as a source of data on health care utilisation [16]. The study used the same CMM protocol as the one used in Croatia. Beyond the equivalent care protocols, both the CMM service in Croatia and in US were offered to patient populations diagnosed with hypertension and at least one additional established cardiovascular disease (CVD) and/or type 2 diabetes mellitus (DMT2) who used five or more medicines daily, similar in terms of sociodemographic and other clinical characteristics (Comparable in terms of number of medical conditions, number of medications at baseline, number and type of drug therapy problems, and gender) with the exception of beneficiary age. Unlike in Croatia where the mean beneficiary age was 72.4 ± 4.6 (range 65–80), the US patient population included a much broader age distribution (21 to 102 years) with 55.5% of patients younger than age 65 years. Regardless of the age disparities between the two patient samples, all the other relevant results, such as type and incidence of drug therapy problems and clinical outcomes (percentage of reduction of blood pressure, glycated haemoglobin, and lipid status) coincide, pointing to the conclusion that these data can be used in our calculations and that the age difference does not influence the study results in a prohibitive manner.

Leading international treatment guidelines for management of hypertension in the adult European population, the European Society of Cardiology and European Society of Hypertension’s guidelines [44] were used as sources of benefits of achieving particular therapeutic goals. Finally, different empirical studies were used as the sources of the incidence rates of particular unwanted clinical events [45,46,47,48,49,50,51,52,53,54,55,56,57,58,59,60,61,62], as explained below. Once the studies reporting the incidence rates of particular unwanted clinical events were identified in the literature, these were discussed with key opinion leaders and experts to confirm their usefulness in the Croatian context and used in our BIA models when deemed relevant. 

### 2.2. Budget Impact Model

**Figure 1:**
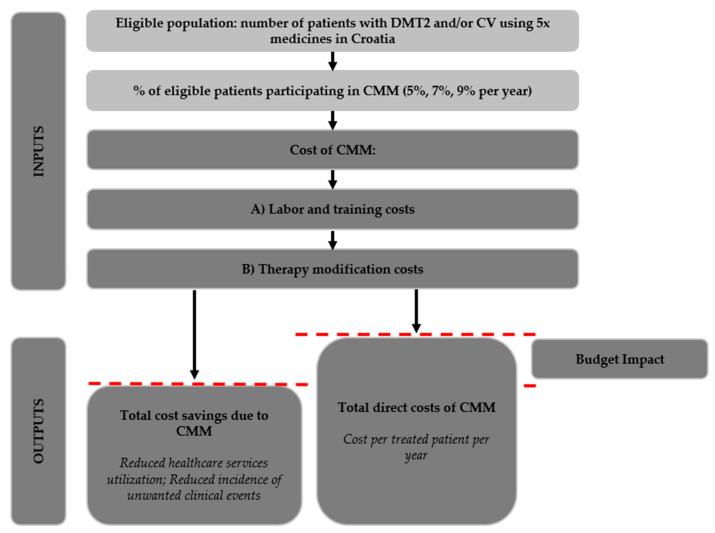
Model structure.

The BIA model, developed in Microsoft Excel (Microsoft Corp., Redmond, WA, USA), is presented in Figure 1. In the status quo scenario (i.e., the current standard of care), eligible patients receive usual primary care (i.e., medication prescribing and consultations in the outpatient setting) with no additional pharmacist-led services explaining why CMM is treated as an addition to the existing standard of care and the status quo is not modelled. The CMM scenario includes inputs and outputs. Model inputs include the eligible population size per year and the total yearly costs of implementing CMM in that population (labour and training costs as well as therapy modification costs). Based on our pilot CMM intervention, we knew beforehand that CMM in Croatia will likely lead to an increase in therapy costs instead of cost savings (as has been observed in other CMM programmes [34]), so we attributed those to the model input parameters (the particulars of the cost and population calculations are explained below). As an output of the model, the BIA compared these total costs of CMM (expressed as aggregate intervention cost per year as well as cost per treated patient per year) to the cost reductions expected from CMM to calculate the budget impact of CMM. These cost reductions (or savings) are based on the reduced incidence of unwanted clinical events and healthcare service utilisation rates due to CMM. As explained in more detail below, the reduced costs of unwanted clinical events and healthcare service utilisation were based on a costing catalogue of CHIF (diagnosis-related group or DRG costs for particular treatment and service) multiplied by the reduction rate of healthcare service utilisation and the reduction rate in the incidence of unwanted clinical events respectively, to obtain the incremental cost savings per patient participating in CMM. Further calculation details are provided in the following section. 

### 2.3. Eligible Patients

Eligible patients were those diagnosed with hypertension and at least one additional established cardiovascular disease (CVD) and/or type 2 diabetes mellitus (DMT2) and using five or more medicines daily (as is typically the case with CMM service [28]). To correct for the fact that some patients have both DMT2 and CVD, we used a simple assumption that all patients with diabetes have a CVD, while the remaining CVD patients do not have DMT2, thereby reducing the total number of prevalent DMT2 + CVD patients taking medication by the number of DMT2 patients (Table 2). Due to fiscal limitations as well as a limited number of trained pharmacists available in the labour market, a nationwide CMM could not be rolled out and offered to all eligible patients in Croatia. Based on the estimated availability of pharmacists (A limited number of clinical pharmacists is currently available rendering it difficult to recruit the required number of professionals (or staff members) to provide the service, without installing additional education) and the maximum number of patient visits per pharmacist per day, as discussed below, we calculated a manageable proportion of eligible patients who could be enrolled in CMM in Croatia each year (Point: BIA does not account for geographical distribution of CMM, availability or cost—taken one national average) (5%, 7%, and 9% respectively each year; Table 2) and used those estimates in the BIA model.

### 2.4. Estimating the Costs of CMM

The Direct Costs of CMM consist of:

(a) Labour and training costs: pharmacists need to be hired and trained to provide CMM (employed by local primary care Health Centres but funded by CHIF). The number of new pharmacists required for CMM was estimated based on the projected number of pharmacists available on the labour market in 2022–2024, the pace at which training can be provided within a single year, the number of working days per year, and the target number of patient visits per day/per pharmacist (To establish a financially viable practice, that is to build a stable revenue base, care needs to be provided to a minimum of 10 to 15 patients per day [64,65]) (Table 3).
(b) Therapy modification costs: based on the results of our pilot study [40] (confirmed by other studies [16,20,34]), the main drug therapy problems typically identified and addressed by CMM pharmacists are the need for additional drug therapy and subtherapeutic dosage [64]. Introducing new medicines and/or increasing dosages creates additional costs for the healthcare system. Based on the findings from our pilot study, we estimated the additional cost per defined daily dose (DDD) at EUR 0.10 per patient in CMM; the total costs of additional medication therapy are presented in the Results section. For each patient involved in our pilot study, the additional medication costs were calculated by subtracting the costs of medication per day (i.e., the sum of costs per prescribed and used daily dose for each drug, obtained from the CHIF) at the last visit from the costs of medication per day at the initial visit. The average was then used to determine the approximate average additional cost of EUR 0.10 per defined daily dose per patient (EUR 37.47 per patient per year in DDD).

### 2.5. Estimating the Cost Savings of CMM

Research has shown that CMM reduces the use of healthcare services and the incidence of unwanted clinical events [16,32,35,65]. However, as already noted, our pilot CMM data are not comprehensive or mature enough to calculate the rates of reductions in the use of healthcare services and the incidence of unwanted clinical events. Hence, we relied on published sources to proxy CMM’s cost-saving effects, as follows:

(a) The rates of reduction in healthcare service utilisation were approximated by dividing the number of avoided healthcare services by the number of patients visits reported in a large-scale study of the effects of CMM in the US (Table 4) [16]. The estimated rates of services avoided per visit obtained from the study of Ramalho de Oliveira et al. were then applied to patient visits within CMM in Croatia to approximate the expected number of avoided healthcare services per visit due to CMM in Croatia. Next, the number of avoided services due to CMM were multiplied by the respective DRG-based prices of service in Croatia to calculate the cost off-setting impact of CMM (Table 4). The BIA also accounted for the cost of employee work days saved because in Croatia, the costs of employment health-related benefits are funded from the CHIF’s budget and hence are relevant from the payer’s perspective.
(b) The rates of reduction in the incidence of unwanted clinical events per patient (Table 5) (While the rates of healthcare service utilisation were calculated per visit (because that is how Ramalho de Oliveira et al. [16] reported their results), the reduction in the incidence rates was calculated per patient (not per visit) since incidence rates are usually reported in such a manner) were based on (1) incidence rates of unwanted clinical events per 1000 inhabitants in two disease groups (CV and CV + DMT2), converted to per patient rates, and reported in various published studies (final column in Table 5) and (2) well-documented target of medication management [44] for all eligible patients participating in CMM, that is, the reduction in blood pressure (SBP for 10 mmHg or DBP for 5 mmHg), since all the patients had at least hypertension as a CVD indication. If patients achieve this target in CMM, we assumed it will lead to a certain percentage reduction in the individual risk of an unwanted clinical effect, as it has been shown in the literature [44].

We estimated the incidence rates of unwanted clinical events per patient and per disease group (DMT2 and CVD) from the published literature (Croatian data were not available so international references were used, Table 5, column 1). From these multisource incidence rates per 1000 inhabitants for a particular event per disease group, we recalculated individual risks of each unwanted event by disease group (column 2). As suggested by the Guidelines for the management of arterial hypertension [44], we assumed these individual risks would be reduced by a certain percentage when the target reduction in blood pressure was reached with CMM (SBP for 10 mmHg or DBP for 5 mmHg, column 3). That is, we assumed that the risk reductions can be achieved once the target reduction in SBP or DBP has been achieved. Based on the results of our pilot study, however, we evaluated that the target reduction in blood pressure (SBP for 10 mmHg or DBP for 5 mmHg) will not be reached in all CMM patients. Instead, we used a more conservative target (lowering SBP by 9 mmHg or DBP by 5 mmHg, based on average reduction actually observed in our pilot study (Ongoing study; to be published). We assumed that this 10% decrease in efficiency of CMM in Croatia (i.e., SBP reduced by 9 mmHg instead of 10 mmHg) will consequently reflect linearly in the 10% reduction in the individual risk reduction (column 4) and incidence rates converted to individual risk rates (column 5 and 6) in all patients participating in CMM.

The cost savings stemming from the reduced incidence rate of unwanted clinical events are DRG-based (Table 6). The costs of treating unwanted clinical events consist of a DRG-based inpatient treatment cost and the cost of rehabilitation following the event (plus the cost of electro stimulator implantation following heart failure). Based on expert opinion, the cost of rehabilitation was assigned to each event to proxy a multitude of possible additional inpatient and outpatient costs surrounding each of the unwanted clinical events of interest, to avoid underestimating the costs of treatments if those were based only on inpatient DRG costs. Due to the lack of more detailed healthcare cost data in Croatia, it was impossible to obtain an average total cost per event (which would include, among other costs, the DRG-based inpatient cost of treatment). Hence, we use the cost of 21-day rehabilitation (which would typically be prescribed to patients in those conditions, based on expert opinion) as a proxy for all the additional costs surrounding each event. To avoid overestimating the costs, on the other hand, we used a conservative cost of rehabilitation at EUR 56.00/day (EUR 1,176,00/21 days, which is an underestimation of the real cost of rehabilitation since under the costing regimen of CHIF, this amount covers only the accommodation in a rehabilitation facility, without physical or any other form of therapy).

**Table 5:** Risk reduction and incidence rates of unwanted clinical events.

Event	Patient Group	Incidence Rate (Per 1000 Inhabitants)	Individual Risk	Individual Risk Reduction *	Individual Risk Reduction (−10%) *	Incidence Rate (Per 1000 Inhabitants)	Individual Risk	Reduction in Individual Risk Due to CMM	Ref
		1	2	3	4	5	6	6–2	
		before intervention	before intervention			after intervention	after intervention		
Heart failure	DMT2 + CVD	23.86	0.02386	40%	36%	15.27	0.01527	0.00859	[45,46,47,48]
	CVD	9.70	0.00970	40%	36%	6.21	0.00621	0.00349	[47,48,49,50,51]
Stroke	DMT2 + CVD	14.60	0.01460	35%	32%	10.00	0.01000	0.00460	[47,48,49,52,53]
	CVD	7.70	0.00770	35%	32%	5.27	0.00527	0.00243	[47,49]
Myocardial infarction—fatal	DMT2 + CVD	18.00	0.01800	20%	18%	14.76	0.01476	0.00324	[47,54]
	CVD	8.70	0.00870	20%	18%	7.13	0.00713	0.00157	[47,55]
Myocardial infarction—nonfatal	DMT2 + CVD	27.8	0.02780	20%	18%	22.80	0.02280	0.00500	[56]
	CVD	13.00	0.01300	20%	18%	10.66	0.01066	0.00234	[57,58]
Angina	DMT2 + CVD	21.60	0.02160	20%	18%	17.71	0.01771	0.00389	[59]
	CVD	14.60	0.01460	20%	18%	11.97	0.01197	0.00263	[60]
Revascularization—stenotic coronary arteries	DMT2 + CVD	3.85	0.00385	20%	18%	3.16	0.00316	0.00069	[59,61]
	CVD	3.85	0.00385	20%	18%	3.16	0.00316	0.00069	[60,61]

* Note: The incidence rates (columns 1 and 5) were divided by 1000 to obtain individual risk rates (columns 2 and 6). As suggested by the Guidelines for the management of arterial hypertension [44], achieving target reduction in blood pressure (lowering SBP by 9 mmHg or DBP by 5 mmHg) will lead to a reduction in the individual risk (by percentage outlined in column 4) and consequently to lower incidence rates converted to individual risk rates (columns 5 and 6) in all patients participating in CMM.

### 2.6. Sensitivity Analysis

Two-way deterministic sensitivity analysis was used to evaluate the impact of overestimating and underestimating the main variable inputs on CMM’s budget impact. Not all input parameters can be considered variable, i.e., some costs are set by CHIF (e.g., DRG costs), some are defined by the availability of the pharmacists in the labour market in Croatia, and some are based on the available Croatian epidemiological data (e.g., prevalence of CVD or DMT2), which means those inputs are predefined and exogenically set. The more interesting variability to investigate concerns the remaining two main parameters: the rates of healthcare services avoided (Table 7) as well as the risk reduction of unwanted clinical events (Table 8). The baseline sensitivity analysis scenario involves 5% over- and underestimation of the rates of healthcare services avoided and the risk reduction of unwanted clinical events. Beyond the baseline scenario, we also explored the impact of a much larger overestimation of the risk reductions and utilisation rates (by 20% and 40%) reported in the original studies [16] used to populate the BIA model (Table 7 and 8). If those rates are overly optimistic and could not be—for whatever reason—achieved in the Croatian context, we used the one-way sensitivity analysis scenario to estimate the budget impact of such a large overestimation of CMM’s benefits, using arbitrary albeit considerably lower reductions in utilisation and risk rates. 

## 3. Results

Total direct costs (Table 9) of labour and training amount to EUR 2,667,098 for 3 years. CMM is expected to increase the cost of medication prescribed to patients by EUR 5,182,864 in 3 years, amounting to the total CMM costs of EUR 7,849,962 for 138,308 patients over 3 years. CMM’s cost per treated patient per year is therefore EUR 57. The annual cost increase is driven by the increase in the patient population covered by CMM (5% in year 1; 7% in year 2; and 9% in year 3) as well as the predicted 1% rise in the prevalence of CVD and DMT2 (Table 2). 

CMM is expected to reduce the utilisation rates and costs of healthcare service utilisation (Table 10) and the incidence of unwanted clinical events (Table 11), leading to a total 3-year reduction in healthcare costs of EUR 7,787,765.60. Given the total CMM costs of EUR 7,849,962, CMM’s 3-year budget impact equals EUR 92,869. Per treated patient incremental cost of CMM is therefore EUR 0.67. 

Based on the incidence rates and the reduced individual risk rates due to CMM for a given event per disease group (Table 5), CMM’s benefits can also be expressed in terms of the number of avoided unwanted clinical events. The number of avoided events per year in the group of patients participating in CMM are presented in Table 12, totalling 2742 cases over 3 years (Some double counting may arise. The number of avoided events was calculated as an individual risk rate for any and all individuals in the sample but some individuals probably face a risk of developing two or more conditions simultaneously. The risk rates of combined conditions are not known). In preventing other severe conditions (stroke, nonfatal myocardial infarction, and others), CMM can contribute to saving and prolonging lives as well as increasing the quality of life and productivity of patients and their caregivers. 

In the sensitivity analysis, we investigated CMM’s budget impact of the rates of avoided healthcare services as well as the risk reduction of unwanted events in case these were 5% under- or overestimated as well as 20% and 40% overestimated (Table 13). Relative to the baseline budget impact estimates, the combined effect of 40% overestimation of both rates yields a budget impact of EUR 3.2 million and the incremental cost per patient of EUR 23. Alternatively, if the benefits of CMM are underestimated by mere 5%, the budget impact would be negative, making CMM the dominant intervention. 

## 4. Discussion

CMM—as modelled in our budget impact analysis—is a large-scale intervention that would encompass over 138,000 patients over 3 years and employ 41 new pharmacists. CMM’s net budget impact—at just over EUR 92,000 for 3 years and EUR 0.67 incremental cost per patient—can be considered modest. That said, CMM appears to be a good investment also because of Croatia’s health and healthcare system profile. As already mentioned, ischaemic heart disease and stroke are the two main causes of death in Croatia, with preventable mortality rates from ischaemic heart disease and stroke twice the EU average [39]. Mortality rates from diabetes have increased sharply since 2000. The rise in mortality from treatable conditions such as diabetes should be a cause for concern and an argument for introducing CMM services, which can help patients and physicians achieve desired health outcomes more efficiently. 

There are various arguments for introducing CMM services into our healthcare systems. CMM is an intersectoral programme, requiring the coordination of GPs and pharmacists. As such, CMM could contribute to strengthening otherwise weak intersectoral policies and contribute to addressing key determinants of ill health, which in turn contribute to high rates of death from preventable and treatable causes. Moreover, CMM can help healthcare payers throughout Europe improve the postlisting value-for-money of prescription medicines. The fact that prescription medicine volumes are rising throughout Europe is not necessarily surprising given our aging populations, but this fact emphasises the need to further promote rational use. Greater efforts need to be made to ensure that medications are appropriately prescribed and coordinated to avoid DTPs, namely omissions, duplicate prescriptions, and harmful interactions, and CMM can be a great asset. As mentioned before, CMM can be used as a basis for developing increasingly detailed prescribing guidelines to monitor and enforce rational medicine use, which would have a double effect: fewer adverse events and lower overall prescribing costs.According to the results of our BIA model, the pilot CMM study could be transformed into a nationwide CMM service in Croatia, at a relatively modest price tag. CMM is not necessarily a dominant intervention in the sense that it reduces costs and generates incremental benefits, but these incremental benefits are generated at a modest cost. However, for CMM to become a reality in Croatia and elsewhere, both the policymakers and the payers need to support the development and implementation of CMM by reimbursing it and making it reproducible and sustainable over time. There need to be governments and health plans willing to support clinical pharmacists, namely professionals eager and capable to provide this service, as CMM will only live its full potential when we have well trained and experienced practitioners. The service is currently being piloted in Croatia although the pharmacists providing it are not being remunerated for their efforts. Considering the fact that CMM seems economically viable, both through this and previous analyses [34], CMM is a highly recommended solution for addressing medication mismanagement and irrational drug use and as such should be a top priority for implementation in the healthcare system.

### Limitations

The first point we wish to address is the realistic representation of healthcare costs. As explained in the methods section, the cost savings of reduced incidence of treating unwanted events are DRG-based. In Croatia, the price of DRGs is considerably lower than in neighbouring EU member states, and their price fluctuates often, depending on the financial situation in the healthcare system [66,67]. Moreover, the DRGs contain only inpatient costs, while the treatment of conditions such as stroke requires additional medications, rehabilitation, and many other (direct and indirect) follow-up costs, which are not considered in the hospital-based DRG. There is no national costing catalogue. To correct (at least partly) for the underestimation of the total DRG-based inpatient cost of the treatment of unwanted clinical events, we added the cost of one-time rehabilitation lasting 21 days to all events although at a fraction of its price. Hence, it is reasonable to expect that the inclusion of all costs of treatments would lead to higher cost savings related to CMM and consequently lower its budget impact. However, even our conservative estimate of the cost-saving impact of CMM shows that CMM can be affordable, even at unrealistically low costs for treating expensive conditions.

The second point we wish to address is the issue of using data from published sources. One may argue that the risk rates and the utilisation rates employed in our analysis—although taken from published sources—may not necessarily be the best representation of the Croatian epidemiological, clinical, and utilisation data. Ideally, we would calculate the actual and detailed costs of treating unwanted clinical events and the costs of particular healthcare services in the Croatian population and multiply those by the actual rates of healthcare services used and the incidence rates of unwanted clinical events per patient group receiving CMM and a group not receiving CMM (and subtract the difference). However, the rate of healthcare service utilisation and incidence rates of unwanted clinical events relevant for our study are not available in Croatia (let alone, for the target patient group). To reduce the risk associated with using published results in our BIA model, several measures were taken. With regard to the utilisation and clinical event rates, we used the study, which was methodologically and results-wise comparable to our CMM study, using the same CMM protocol as the one used in Croatia and a comparable patient population [16]. With respect to the incidence rates of unwanted clinical events [45,46,47,48,49,50,51,52,53,54,55,56,57,58,59,60,61,62], these were discussed with key opinion leaders to confirm their applicability in the Croatian context. Finally, the sensitivity analysis was developed precisely to test the effect of overestimating the individual risk and rates of utilisation, to obtain a sense of the effect of over- or underestimation of these parameters as possible consequences of using data from different sources. Under the unfavourable assumption of 40% overestimation of the risk and utilisation rate reductions due to CMM, the budget impact reaches around EUR 3.2 mil for 3 years. Nevertheless, even with this relatively high budget impact, when we take into account the large number of patients included in CMM, the incremental cost per patient remains relatively low. 

Third, the usefulness of data from the US and its transferability to the Croatian context may be hampered by the differences in healthcare payments and health insurance coverage (and the related accessibility of CMM). The United States has Medicare, a government-provided insurance for older individuals compared to more private insurance options for younger individuals. The coverage differences result in varying use of health care services. Unlike the US, Croatia operates a generous universal health insurance covering all citizens, funded from income-based contributions. Healthcare is free at the point of entry, except for certain medicines which require copayments. Hence, there is little variability in age-related access and use of healthcare, which would be determined by insurance coverage since healthcare is accessible and free at the point of entry for all (including CMM). In that sense, the US data may offer a conservative outlook on CMM’s benefits relative to its potential in Croatia. 

The fourth point we wish to address is the scope of the BIA model. CMM is an intervention that could be intended for all patient groups irrespective of the condition they suffer from. The BIA conducted in this study included CVD and DMT2 patients only, rendering the budget impact relevant exclusively for this patient group. Future research should be focused on evaluating the impact of CMM on a broader range of health conditions. 

Finally, the implementation of CMM initially leads to medication cost increase, as the main drug therapy problems typically identified and addressed by CMM service are the need for additional drug therapy and subtherapeutic dosage requiring increasing the doses and introducing new therapies. However, in the course of time, most often within the first year of CMM introduction, the related cost savings resulting from the reduction in the use of healthcare services and the incidence of unwanted clinical events balance and exceed this initial cost increase. 

## 5. Conclusions

CMM provided by trained pharmacists reduces the unnecessary and often harmful use of medications and can help patients and physicians achieve desired health outcomes more efficiently. The budget impact analysis performed in our study shows that CMM services for high-risk patients led to a budget impact at just over EUR 92,000 within a 3-year horizon, rendering CMM an affordable intervention. Studies quantifying the costs and the effects of pharmacist interventions are lacking and lag behind other public health interventions and technologies. In the era of increasing and irrational medicine use, medication errors, inappropriate prescribing, duplicate therapy, and detrimental interactions on the one hand and tight healthcare budgets on the other, we cannot afford to ignore the costs and benefits of pharmacist interventions nor their potential to increase the value of money spent on medicines. 

## Figures and Tables

**Table 1:** Standardised CMM activities in the patient care process [22].

The Patient Care Process	
ASSESSMENT OF THE PATIENT’S DRUG-RELATED NEEDS	➢ Meet the patient and understand patient’s medication experience (preferences, expectations, and beliefs). ➢ Collect patient-specific information: demographics, health-related behaviour (alcohol, tobacco, and caffeine intake) and clinical information (relevant medical history, medication history, current medication list including prescription and over-the-counter medications, herbal remedies, supplements and medications used for a limited period of time, and relevant laboratory values) including allergies, side effects, and immunizations. ➢ Prioritise patient’s active medical conditions and medication-related needs.
IDENTIFICATION OF DRUG-RELATED PROBLEMS	➢ Determine that all the patient’s medications are properly indicated, the most effective given the medical condition, the safest possible, and that the patient is able and willing to take the medication as intended. ➢ Analyse the assessment data to determine if any drug therapy problems are present.
CARE PLAN DEVELOPMENT	➢ Identify therapy goals for each indication managed with drug therapy. ➢ Develop a care plan that includes interventions to resolve current drug therapy problems, prevent potential drug therapy problems, and achieve therapy goals. ➢ Discuss and negotiate the care plan with the patient and his prescriber, ensure patient’s and prescriber’s understanding and agreement with the plan, and schedule follow-up evaluation. ➢ Document the care plan, which includes all the steps and clinical status determined for every patient’s medical condition.
FOLLOW-UP EVALUATION	➢ Follow-up evaluation for each patient reassesses whether any new drug therapy problems have developed, monitors patient’s progress toward the achievement of the goals of therapy, and refines the care plan to ensure therapy goals are achieved and medication therapy is optimised.

**Table 2:** Eligible population and the number of patients in CMM.

Croatian Population	4,087,934			Source
	2022	2023	2024	
DMT2 prevalence (%)	7.74%	7.82%	7.89%	Prevalence growth is estimated at 1% yearly [62]
Of which on medication	76%	76%	76%	[38]
Number of DMT2 patients on medication	240,398	242,802	245,230	
CVD prevalence (%)	26.02%	26.28%	26.54%	Prevalence growth is estimated at 1% yearly [63].
Of which on medication	90%	90%	90%	Data based on expert opinion
Number of CVD patients on medication	765,687	773,344	781,077	Equal to the number of prevalent patients DMT2 + CVD on medication in Croatia *
Of which on 5+ medicines	85%	85%	85%	Data based on expert opinion and pilot results
Total number of CMM eligible patients in Croatia, of which:	650,834	657,342	663,916	
DMT2 + CVD	204,340	206,383	208,447	
CVD	446,494	450,959	455,469	
% of eligible patients included in CMM	5.0%	7.0%	9.0%	Calculated based on the number of eligible trained pharmacists in the labour market
Number of eligible patients included in CMM, of which:	32,542	46,014	59,752	
DMT2 + CVD	10,217	14,447	18,760	
CVD	22,325	31,567	40,992	

* Corrected for CVD/DMT2 overlap.

**Table 3:** Eligible population and the number of patients in CMM.

Cost Per Pharmacist			2022	2023	2024
Number of patient visits per day	11	Number of pharmacists in CMM	22	32	41
Working days per month/year	22/264	Total cost of labour/year	EUR 632,008.13	EUR 889,177.20	EUR1,154,142.67
Number of visits month/year	264/2904	Total cost of training/year *	EUR 4482.27	EUR 1855.73	EUR 1892.40
Before tax salary pharmacist + administrative personnel */year	EUR 28,000.00				
Average cost per visit	EUR 9.60				

Note: Half of the administrative personnel’s salary was attributed to each pharmacist (one administrative person was assumed to serve two pharmacists). Because the budget impact analysis uses a short-term time horizon and overhead costs are fixed in the short term, these overhead costs are ordinarily excluded from BIA. * Estimated at EUR 200 per pharmacists.

**Table 4:** Rates of avoided healthcare service utilisation and their respective costs in Croatia.

Data from Ramalho de Oliveira et al. (2010) [16]				
Healthcare services	Total number of encounters in CMM	Total number of healthcare services avoided	Rate of services avoided, per visit	DRG-based price of services in Croatia **
Clinic outpatient visit avoided	33,706	7219.1 *	0.214	EUR 10.40
Specialty office visit avoided	33,706	1346	0.040	EUR 18.93
Employee work days saved	33,706	277	0.008	EUR 47.19
Laboratory service avoided	33,706	240	0.007	EUR 8.10
Urgent care visit avoided	33,706	355	0.011	EUR 54.13
Hospital admission avoided	33,706	41	0.001	EUR 120.13
Nursing home admissions	33,706	3	0.000	EUR 20.00
Home health visit	33,706	1	0.000	EUR 16.02

Note: * The rate of clinic outpatient visit avoided was reduced by 30% relative to the original study (which reported 10,313 services avoided) to account for the fact that pharmacists in Croatia, unlike in the US, cannot prescribe medicines and hence patients still need to visit the primary care physician to obtain prescriptions. A total of 30% is an estimate based on an assumption that in some instances (at least one third of GP encounters) patients will still need to visit their GP to have their therapy modified, whereas in the rest of the occurrences where GPs have established direct rapport with practising pharmacists, GPs would adopt pharmacist recommendations and alter patient therapies without seeing the patient ** available at https://hzzo.hr/hzzo-za-partnere/sifrarnici-hzzo-0 (access date 4 February 2022).

**Table 6:** DRG prices for the treatment of unwanted clinical events (costing catalogue of CHIF).

Event	DRG-Based Price (InPatient Treatment)	DRG-Based Price of the Follow-Up Treatment and/or Rehabilitation	Total Cost of Event Treatment
Heart failure	EUR 1182.24	EUR 1176.00 + EUR 2473.95 (pacemaker)	EUR 4832.19
Stroke	EUR 1959.45	EUR 1176.00	EUR 3135.45
Myocardial infarction—fatal	EUR 864.79	EUR 1176.00	EUR 864.79
Myocardial infarction—nonfatal	EUR 1806.20	EUR 1176.00	EUR 2982.20
Angina	EUR 1127.51	EUR 1176.00	EUR 2303.51
Revascularization—stenotic coronary arteries	EUR 1061.83	EUR 1176.00	EUR 2237.83

Note: DRG prices from the costing catalogue of CHIF available at https://hzzo.hr/hzzo-za-partnere/sifrarnici-hzzo-0 (access date 14 February 2022).

**Table 7:** Sensitivity analysis—rates of avoided healthcare services.

Healthcare Services	Baseline Rate of Services Avoided, Per Visit	+5%	−5%	−20%	−40%
Clinic outpatient visit avoided	0.214	0.042	0.203	0.171	0.129
Specialty office visit avoided	0.040	0.009	0.038	0.032	0.024
Employee work days saved	0.008	0.007	0.008	0.007	0.005
Laboratory service avoided	0.007	0.011	0.007	0.006	0.004
Urgent care visit avoided	0.011	0.001	0.010	0.008	0.006
Hospital admission avoided	0.001	0.000	0.001	0.001	0.001
Nursing home admissions	0.000	0.000	0.000	0.000	0.000
Home health visit	0.000	0.042	0.000	0.000	0.000

**Table 8:** Sensitivity analysis—risk reduction of unwanted clinical events.

Event	Patient Group	Baseline Individual Risk Reduction Due to CMM	+5%	−5%	−20%	−40%
Heart failure	DMT2 + CVD	0.00859	0.00902	0.00816	0.00687	0.00515
	CVD	0.00349	0.00367	0.00332	0.00279	0.00210
Stroke	DMT2 + CVD	0.00460	0.00483	0.00437	0.00368	0.00276
	CVD	0.00243	0.00255	0.00230	0.00194	0.00146
Myocardial infarction—fatal	DMT2 + CVD	0.00324	0.00340	0.00308	0.00259	0.00194
	CVD	0.00157	0.00164	0.00149	0.00125	0.00094
Myocardial infarction—nonfatal	DMT2 + CVD	0.00500	0.00525	0.00475	0.00400	0.00300
	CVD	0.00234	0.00246	0.00222	0.00187	0.00140
Angina	DMT2 + CVD	0.00389	0.00408	0.00369	0.00311	0.00233
	CVD	0.00263	0.00276	0.00250	0.00210	0.00158
Revascularization—stenotic coronary arteries	DMT2 + CVD	0.00069	0.00073	0.00066	0.00055	0.00042
	CVD	0.00069	0.00073	0.00066	0.00055	0.00042

**Table 9:** Total costs of CMM in Croatia.

Total Direct Costs	2022	2023	2024	Total 2022–2024
Labour costs + education/training costs	EUR 627,526 (22 pharmacists)	EUR 887,321 (32 pharmacists)	EUR 1,152,250 (41 pharmacists)	EUR 2,667,098
Additional medication therapy cost	EUR 1,219,446	EUR 1,724,296	EUR 2,239,122	EUR 5,182,864
Total	EUR 1,846,972	EUR 2,611,618	EUR 3,391,372	EUR 7,849,962

**Table 10:** Cost savings: reduced healthcare service utilisation.

Cost Savings: Reduced Healthcare Service Utilisation	2022	2023	2024	Total 2022–2024
Clinic outpatient visits avoided	EUR 144,970	EUR 204,988	EUR 266,192	EUR 616,150
Specialty office visit avoided	EUR 49,204	EUR 69,575	EUR 90,348	EUR 209,128
Employee work days saved	EUR 25,241	EUR 35,691	EUR 46,348	EUR 107,280
Laboratory service avoided	EUR 3753	EUR 5307	EUR 6891	EUR 15,951
Urgent care visit avoided	EUR 37,107	EUR 52,469	EUR 68,135	EUR 157,712
Hospital admission avoided	EUR 9511	EUR 13,448	EUR 17,463	EUR 40,422
Nursing home admissions	EUR 116	EUR 164	EUR 213	EUR 492
Home health visit	EUR 31	EUR 44	EUR 57	EUR 131
Total	EUR 269,934	EUR 381,686	EUR 495,647	EUR 1,147,267

Note: Data not available per disease group.

**Table 11:** Cost savings: reduced incidence of unwanted clinical events.

	Patient Group	2022	2023	2024	Total 2022–2024
Heart failure	DMT2 + CVD	EUR 424,072	EUR 599,637	EUR 778,672	EUR 1,802,380
	CVD	EUR 376,707	EUR 532,664	EUR 691,702	EUR 1,601,073
Stroke	DMT2 + CVD	EUR 147,328	EUR 208,322	EUR 270,521	EUR 626,172
	CVD	EUR 321,921	EUR 455,197	EUR 591,105	EUR 1,368,224
Myocardial infarction—fatal	DMT2 + CVD	EUR 28,627	EUR 40,479	EUR 52,564	EUR 121,670
	CVD	EUR 62,552	EUR 88,448	EUR 114,856	EUR 265,857
Myocardial infarction—nonfatal	DMT2 + CVD	EUR 44,213	EUR 62,517	EUR 81,183	EUR 187,913
	CVD	EUR 45,176	EUR 63,879	EUR 82,952	EUR 192,007
Angina	DMT2 + CVD	EUR 34,352	EUR 48,574	EUR 63,077	EUR 146,004
	CVD	EUR 50,736	EUR 71,741	EUR 93,161	EUR 215,639
Revascularization—stenotic coronary arteries	DMT2 + CVD	EUR 6123	EUR 8658	EUR 11,243	EUR 26,024
	CVD	EUR 13,379	EUR 18,918	EUR 24,567	EUR 56,864
Total		EUR 1,555,187	EUR 2,199,035	EUR 2,855,604	EUR 6,609,827

**Table 12:** Number of avoided unwanted events in the eligible population (*n* = 138,308).

		2022	2023	2024	Total 2022–2024
Heart failure	DMT2 + CVD	88	124	161	373
	CVD	78	110	143	331
Stroke	DMT2 + CVD	47	66	86	200
	CVD	103	145	189	436
Myocardial infarction—fatal	DMT2 + CVD	33	47	61	141
	CVD	72	102	133	307
Myocardial infarction—nonfatal	DMT2 + CVD	51	72	94	217
	CVD	52	74	96	222
Angina	DMT2 + CVD	40	56	73	169
	CVD	59	83	108	249
Revascularization—stenotic coronary arteries	DMT2 + CVD	7	10	13	30
	CVD	15	22	28	66
Total		645	912	1185	2742

**Table 13:** Sensitivity analysis—risk reduction of unwanted clinical events and the rates of avoided healthcare services.

	Baseline	+5%	−5%	−20%	−40%
Total budget impact for 3 years	EUR 92,869	− EUR 294,986	EUR 480,723	EUR 1,644,287	EUR 3,195,706
Incremental cost per treated patient per year	EUR 0.67	− EUR 2	EUR 3	EUR 12	EUR 23

## Data Availability

The leading author has full access to all the data in the study. Data sharing is available upon request.
